# Social characteristics associated with disparities in smoking rates in Israel

**DOI:** 10.1186/s13584-016-0095-2

**Published:** 2016-12-01

**Authors:** Ofra Kalter-Leibovici, Angela Chetrit, Shlomit Avni, Emma Averbuch, Ilya Novikov, Nihaya Daoud

**Affiliations:** 1 Unit of Cardiovascular Epidemiology, The Gertner Institute for Epidemiology & Health Policy Research, Tel-Hashomer, Israel; 2 Sackler Faculty of Medicine, Tel-Aviv University, Tel-Aviv, Israel; 3 Reduction of Health Inequalities Unit in the Administration for Strategic and Economic Planning at the Israeli Ministry of Health, Jerusalem, Israel; 4 Biostatistics Unit, The Gertner Institute for Epidemiology & Health Policy Research, Tel-Hashomer, Israel; 5 Department of Public Health, Faculty of Health Sciences, Ben-Gurion University of the Negev, Beer-Sheva, Israel

**Keywords:** Cigarette smoking, Smoking uptake, Health disparities, Education, Poverty, Immigrant, Ethnicity, Gender

## Abstract

**Background:**

Cigarette smoking is a major cause of health disparities. We aimed to determine social characteristics associated with smoking status and age at smoking initiation in the ethnically-diverse population of Israel.

**Methods:**

This is a cross-sectional survey, based on data collected during 2010 by the Israel Bureau of Statistics, in a representative nationwide sample of 7,524 adults (≥20 years). Information collected by personal interviews included a broad set of demographic and socio-economic characteristics and detailed information on smoking habits. Associations between social characteristics and smoking habits were tested in multivariable regression models.

**Results:**

Current smoking was more frequent among men than among women (30.9 % vs. 16.8 %; *p* < 0.0001). In multivariable regression analysis, the association of some social characteristics with smoking status differed by gender. Lower socioeconomic status (reflected by higher rate of unemployment, lower income, possession of fewer material assets, difficulty to meet living expenses) and lower educational level were significantly associated with current smoking among men but not among women. Family status other than being married was associated with higher likelihood of being a current smoker, while being traditional or observant was associated with a lower likelihood of ever smoking among both gender groups. Arab minority men and male immigrants from the former Soviet Union countries were more frequently current smokers than Israeli-born Jewish men [adjusted odds ratio (95 % confidence interval): 1.53 (1.22, 1.93) and 1.37 (1.01-1.87), respectively]. Compared to Israeli-born men, the age at smoking initiation was younger among male immigrants, and older among Arab minority men [adjusted hazard ratio (95 % confidence interval): 1.360 (1.165-1.586), and 0.849 (0.749-0.962), respectively]. While the prevalence of current smoking was lower in younger birth cohorts, the age at smoking initiation among ever-smokers declined as well.

**Conclusions:**

Among several subgroups within the Israeli population the smoking uptake is high, e.g. Arab men, men who are less affluent, who have lower educational level, and male immigrants. These subgroups should be prioritized for intervention to reduce the burden of smoking. To be effective, gender, cultural background and socioeconomic characteristics should be considered in the design and implementation of culturally-congruent tobacco control and smoking prevention and cessation interventions.

**Electronic supplementary material:**

The online version of this article (doi:10.1186/s13584-016-0095-2) contains supplementary material, which is available to authorized users.

## Background

Tobacco smoking is associated with increased morbidity and mortality risk due to cardiovascular disease, malignant neoplasms and chronic obstructive lung disease [1]. Men who continue smoking die, on average, 10 years earlier than lifelong non-smokers [2, 3]. In 2014, more than 7,000 deaths in Israel were attributed to smoking [4]. The WHO global burden of disease study suggests that smoking, the single most important risk factor, accounted for 26 % of male deaths and 9 % of female deaths in developed countries [5].

Tobacco smoking, including second-hand smoking, is also a leading factor for global disease burden, accounting for 6.3 % of disability-adjusted life years (DALYs; sum of years lived with disability and years of life lost). In 2010, tobacco smoking accounted for 31 % of global ischemic heart disease DALYs [6].

Smoking is an increasing cause of health inequalities in high-income countries [7]. People of low socioeconomic status (SES) (reflecting low occupational, educational and/or income level), have a significantly higher rate of smoking compared to people of higher SES [8]. Smokers from more economically deprived population subgroups also have higher levels of cigarette consumption and are less likely to be successful when trying to stop smoking [9]. Data suggest that a major proportion of health inequalities associated with social position in men is attributed to smoking [10–12].

In Israel, cigarette smoking is more common among Arab men compared to Jewish men, and while a decline in smoking rates was reported between 2000 and 2008 among Jewish men, an opposite trend was observed among Arab men [13]. Despite high smoking rates, Arab men start smoking at an older age compared to Jewish men [14]. In contrast, immigrants from the former Soviet Union start smoking at a younger age compared to Jews who were born in Israel [14, 15]. Information on a broad set of demographic and socioeconomic characteristics associated with cigarette smoking in the adult population of Israel is missing.

## Methods

We analyzed the dataset of the Social Survey of the Israel Central Bureau of Statistics, conducted in 2010. The survey methods have been previously described [16]. In brief, participants (*n* = 7,524) were non-institutionalized adult Israeli residents (≥20 years old), representing 4.8 million people in this age group. The study sample was based on the Israel population registry and stratified according to five population subgroups [Arabs living in East Jerusalem, Arabs living elsewhere, two immigrant subgroups (immigration year before 1990 and thereafter) and Israeli-born Jews], 7 age groups (20-24, 25-34, 35-44, 45-54, 55-64, 65-74, and 75+ years) and two gender subgroups. The number of people included in each subgroup reflected the relative size of the specific subgroup within the total Israeli population. The study questionnaire included information on demographic, social and economic characteristics, e.g. age, gender, marital and employment status, education, religiosity, income, possession of material assets [i.e. house(s) and car(s)], number of household members, and year of immigration. Detailed information on leisure physical activity, body weight and height, and self-reported health status was obtained. Data on cigarette smoking were obtained from the following set of questions: “Do you smoke cigarettes?” If answered affirmatively, people were asked “How many cigarettes do you smoke per day?” and “At what age did you start smoking?” People who denied smoking in the first question were further asked: “Did you ever smoke cigarettes?” If answered affirmatively, they were subsequently asked about the number of cigarettes they used to smoke per day, the age of smoking initiation and age at smoking cessation. Current or past cigarette smoking was defined as smoking at least one cigarette a day. The consent rate was 83 %.

### Statistical analysis

Because of the large gender differences in smoking habits, analyses were conducted for men and women separately. Associations between participants’ characteristics and smoking status and age at smoking initiation were tested using the chi-square statistic. Bi-variate correlations between the socio-economic, demographic and lifestyle characteristics were tested, using Cramér’s V statistic. The variables were weakly correlated and hence the risk of multi-co-linearity is limited (see Additional file 1: Table S1). Multiple multinomial logistic regression models were fitted to test for characteristics that were significantly and independently associated with smoking status (categorized as current, past or never smoker), with never-smokers serving as a reference group. Bivariate interactions between the model covariates were also tested. We used the Cox proportional hazards model to test for variables associated with the age at smoking initiation. In this analysis age was used as the mean value of the 5-year age category. Among women, the analysis of characteristics associated with the age at smoking initiation was restricted to Jewish women exclusively, since there were only 78 Arab women who reported ever smoking. Because of the large sample size which provided a high level of statistical power, variables entered into the multiple regression models were those found to be significantly associated with smoking status in univariate analyses with p-value <0.05. Information on smoking status was complete and age at smoking initiation was missing for 8 participants. In general, the rate of missing information was very low, except for the following variables: body mass index (BMI) (3.8 %), religiosity (2 %), years of education (1.5 %) and ability to meet living expenses (<1.0 %). The multiple regression models included participants with complete dataset (‘full case analysis’); 95 % male and 90 % female participants were included in the multiple regression analyses of smoking status, and 99 % male and 96 % female participants were included in the multiple regression models of age at smoking initiation.

## Results

Of 7,524 participants (3,687 men and 3,837 women), 59.2 % never smoked, 17.1 % stopped smoking, and 23.7 % were current smokers. Current smoking was more frequent among men than among women (30.9 % vs. 16.8 %), and women were more likely to be never smokers than men (71.6 % vs. 46.2 %); *p* < 0.0001.

**Fig. 1:**
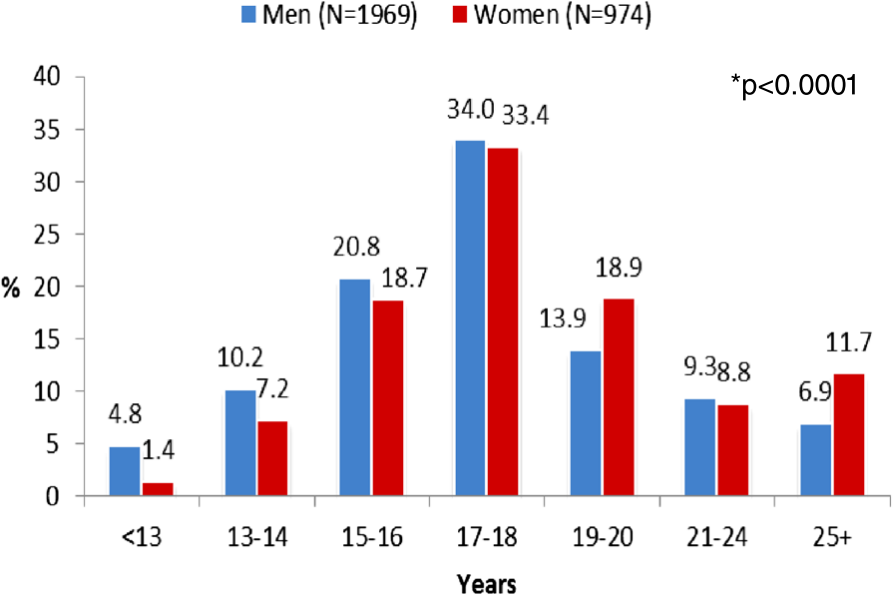
Age at smoking initiation among men and women. * P-value for the association between gender and age at smoking initiation

Men also reported a younger age at smoking initiation than women; 36 % of male ever-smokers (past or current) started smoking before 17 years of age, compared to 27 % of female ever-smokers (Fig. 1; *p* < 0.0001). About one-third of male and female ever-smokers started smoking at the age of 17-18 years.

**Fig. 2:**
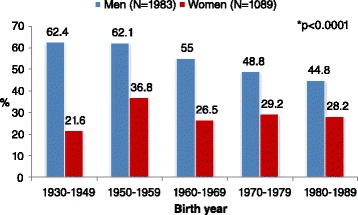
The proportion of ever smokers (past or current) by birth year and gender. * P-value for the association between smoking rates and year of birth, in both gender subgroups

There was a constant decline in the proportion of ever-smokers among men born in 1960 or thereafter. In contrast, there was a transient decline in ever-smoking rates among women who were born in the 60s, but this trend did not continue thereafter (Fig. 2; *p* < 0.0001).

**Fig. 3:**
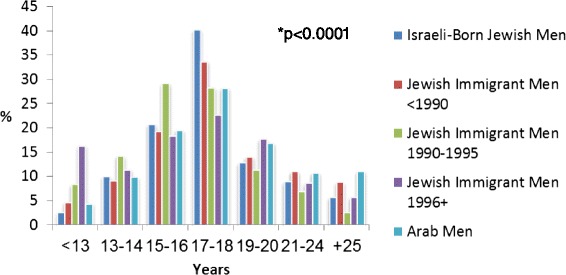
Age at smoking initiation by population subgroup among men. Israeli-born Jewish men (*N* = 876). Jewish immigrant men (*N* = 348). Arab men (*N* = 392). * P-value for the association between population subgroup and age at smoking initiation

Tables 1, 2 and 3 show univariate associations between smoking status and demographic, socioeconomic, and health-related characteristics among men and women. The prevalence of current smoking declined after the age of 54 years in both gender groups, and the rates of past smoking increased with age among men (Table 1; *p* < 0.0001). Current smoking was 1.65-times more prevalent among Arab men than among Jewish men, while Jewish women were twice more likely to report current cigarette smoking than Arab women (Table 1; *p* < 0.0001). Jewish men who immigrated to Israel in 1990 or thereafter had the highest rates of current smoking (Table 1; *p* < 0.0001). Jewish male immigrants differed from Israeli-born Jews also in the age at smoking initiation; 27.5 % of ever-smoking men who immigrated to Israel after 1995 started smoking by the age of 14 years, compared to 12.3 % among Jewish men born in Israel (Fig. 3; *p* < 0.0001). Current smoking was more common among unmarried or divorced men and women. Men with 13 or more years of schooling were less likely to be current smokers and more likely to be never smokers compared to men with lower educational levels. In contrast, women with up to 8 years of schooling had the lowest current smoking and the highest never smoking rates (Table 2; *p* < 0.0001). Unemployment and poverty were associated with higher prevalence rates of current smoking only among men. Men who reported poor health status were more frequently past smokers, while women who reported good health status were more frequently current smokers. Men and women who were current smokers were more likely to have normal body weight (BMI <25 kg/m^2^), while overweight (BMI ≥25 kg/m^2^) was more frequent among male past smokers. Men and women who were current smokers were less likely to report being engaged in leisure physical exercise (Table 3).

**Table 1:** Demographic characteristics associated with cigarette smoking status: univariate analysis

	Men				Women			
	Never *N* = 1,704	Past *N* = 844	Current *N* = 1,139	*P*	Never *N* = 2,748	Past *N* = 444	Current *N* = 645	*P*
Age group, yrs. N, (%)				<0.0001				<0.0001
20-24	262 (59.3)	30 (6.8)	150 (33.9)		336 (76.2)	16 (3.6)	90 (20.4)	
25-34	448 (50.3)	117 (13.2)	325 (36.5)		591 (70.0)	82 (9.7)	171 (20.3)	
35-44	367 (51.9)	110 (15.6)	230 (32.5)		499 (70.8)	85 (12.1)	121 (17.2)	
45-54	232 (38.9)	147 (24.6)	218 (36.5)		402 (67.6)	72 (12.1)	121 (20.3)	
55-64	195 (36.5)	199 (37.3)	140 (26.2)		402 (69.1)	87 (15.0)	93 (16.0)	
65-74	103 (36.9)	123 (44.1)	53 (19.0)		254 (72.6)	61 (17.4)	35 (10.0)	
75+	96 (40.5)	118 (49.8)	23 (9.7)		264 (82.8)	41 (12.9)	14 (4.4)	
Population sub-group; N, (%)				<0.0001				<0.0001
Arabs	279 (41.5)	86 (12.8)	307 (45.7)		600 (88.5)	20 (3.0)	58 (8.6)	
Jews and others:	1,424 (47.3)	758 (25.2)	832 (27.6)		2,147 (68.0)	424 (13.4)	587 (18.6)	
Born in Israel	957 (52.2)	365 (19.9)	511 (27.9)		1176 (65.7)	241 (13.5)	372 (20.8)	
Immigrants before 1990	245 (40.1)	229 (37.5)	137 (22.4)		496 (71.7)	110 (15.9)	86 (12.4)	
Immigrants between 1990 and 1995	149 (42.0)	97 (27.3)	109 (30.7)		285 (69.3)	47 (11.4)	79 (19.2)	
Immigrants after 1995	73 (34.0)	67 (31.1)	75 (34.9)		190 (71.4)	26 (9.8)	50 (18.8)	
Religiosity; N, (%)				<0.0001				<0.0001
Very religious	165 (64.7)	56 (22.0)	34 (13.3)		333 (94.9)	13 (3.7)	5 (1.4)	
Observant	483 (50.1)	201 (20.9)	280 (29.1)		909 (83.6)	69 (6.3)	110 (10.1)	
Traditional	371 (42.6)	191 (22.0)	308 (35.4)		561 (65.6)	103 (12.1)	191 (22.3)	
Secular	649 (42.8)	381 (25.1)	487 (32.1)		895 (61.2)	246 (16.8)	321 (22.0)	
Marital status; N, (%)				<0.0001				<0.0001
Married	1,155 (45.9)	660 (26.2)	702 (27.9)		1,826 (75.6)	282 (11.7)	308 (12.8)	
Divorced	46 (26.6)	47 (27.2)	80 (46.2)		182 (55.8)	54 (16.6)	90 (27.6)	
Widowed	29 (39.7)	29 (39.7)	15 (20.6)		285 (76.0)	49 (13.1)	41 (11.0)	
Unmarried	473 (51.3)	108 (11.7)	341 (37.0)		454 (63.2)	59 (8.2)	205 (28.6)	
Number of people in household: (%)				<0.0001				<0.0001
1	142 (38.7)	84 (22.9)	141 (38.4)		327 (66.6)	75 (15.3)	89 (18.1)	
2	328 (41.6)	264 (33.5)	197 (25.0)		610 (68.3)	133 (14.9)	150 (16.8)	
3	302 (47.6)	151 (23.8)	181 (28.6)		437 (67.1)	76 (11.7)	138 (21.2)	
4	352 (48.4)	144 (19.8)	232 (31.9)		496 (69.6)	84 (11.8)	133 (18.7)	
5+	580 (49.6)	201 (17.2)	388 (33.2)		878 (80.6)	76 (7.0)	135 (12.4)

**Table 2:** Socioeconomic characteristics associated with cigarette smoking status: univariate analysis

	Men				Women			
	Never *N* = 1,704	Past *N* = 844	Current *N* = 1,139	*P*	Never *N* = 2,748	Past *N* = 444	Current *N* = 645	*P*
Years of education; N, (%)				<0.0001				<0.0001
0-8	75 (29.1)	77 (29.8)	106 (41.1)		257 (82.1)	25 (8.0)	31 (9.9)	
9-12	413 (39.1)	195 (18.5)	449 (42.5)		620 (70.4)	78 (8.9)	183 (20.8)	
13+	1,207 (51.4)	563 (24.0)	580 (24.7)		1,792 (70.1)	337 (13.2)	426 (16.7)	
Employment status; N, (%)				<0.0001				<0.0001
Employed	1,221 (46.7)	550 (21.0)	846 (32.3)		1,470 (66.5)	283 (12.8)	458 (20.7)	
Unemployed	53 (31.9)	26 (15.7)	87 (52.4)		129 (77.7)	8 (4.8)	29 (17.5)	
Not included in the labor force	430 (47.6)	268 (29.7)	206 (22.8)		1,149 (78.7)	153 (10.5)	158 (10.8)	
Mean gross income per-capita per month; N, (%)				<0.0001				<0.0001
<2,000 NIS	439 (44.3)	187 (18.9)	365 (36.8)		875 (80.9)	74 (6.8)	133 (12.3)	
2,000-4,000 NIS	427 (45.4)	239 (25.4)	275 (29.2)		746 (70.2)	116 (10.9)	201 (18.9)	
>4,000 NIS	605 (48.5)	323 (25.9)	320 (25.6)		711 (64.2)	188 (17.0)	208 (18.8)	
No information	233 (46.0)	95 (18.7)	179 (35.3)		416 (71.1)	66 (11.3)	103 (17.6)	
	Men (*N* = 3,686)				Women (*N* = 3,837)			
	Never *N* = 1,704	Past *N* = 844	Current *N* = 1,139	*P*	Never *N* = 2,748	Past *N* = 444	Current *N* = 645	*P*
Possession of material assets; N, (%)				<0.0001				<0.0001
Neither car nor house	221 (41.0)	97 (18.0)	221 (41.0)		418 (66.8)	72 (11.5)	136 (21.7)	
Car only	166 (39.6)	82 (19.6)	171 (40.8)		213 (57.7)	60 (16.3)	96 (26.0)	
House only	324 (46.8)	172 (24.8)	197 (28.4)		737 (80.3)	80 (8.7)	101 (11.0)	
A car and a house	650 (48.7)	314 (23.5)	372 (27.8)		931 (74.3)	131 (10.5)	191 (15.2)	
A house and two cars or more	187 (50.8)	79 (21.5)	102 (27.7)		254 (65.8)	49 (12.7)	83 (21.5)	
Two houses or more	156 (47.0)	100 (30.1)	76 (22.9)		195 (68.4)	52 (18.3)	38 (13.3)	
Being successful in meeting living expenses; N, (%)				<0.0001				<0.0001
Highly successful	375 (58.5)	137 (21.4)	129 (20.1)		402 (72.4)	70 (12.6)	83 (15.0)	
Somewhat successful	847 (47.5)	434 (24.3)	504 (28.2)		1,407 (74.2)	221 (11.7)	269 (14.2)	
Not very successful	368 (38.7)	205 (21.6)	377 (39.7)		734 (70.2)	113 (10.8)	199 (19.0)	
Unsuccessful	95 (34.3)	61 (22.0)	121 (43.7)		177 (59.2)	38 (12.7)	84 (13.2)

**Table 3:** Health-related characteristics associated with cigarette smoking status: univariate analysis

Smoking status	Men				Women			
	Never *N* = 1,704	Past *N* = 844	Current *N* = 1,139	*P*	Never *N* = 2,748	Past *N* = 444	Current *N* = 645	*P*
Body mass index (kg/m^2^); N, (%)				<0.0001				0.0002
Normal (<25.0)	758 (48.7)	273 (17.5)	527 (33.8)		1,404 (70.5)	205 (10.3)	384 (19.3)	
Overweight (≥25.0 and <30.0)	675 (44.9)	382 (25.4)	445 (29.6)		728 (71.3)	142 (13.9)	151 (14.8)	
Obese (≥30.0)	235 (42.2)	177 (31.8)	145 (26.0)		456 (74.9)	70 (11.5)	83 (13.6)	
Self-reported leisure physical activity; N, (%)*				<0.0001				0.0012
None	701 (39.9)	391 (22.3)	664 (37.8)		1,578 (72.2)	217 (9.9)	392 (17.9)	
Less than recommended	241 (50.8)	126 (26.6)	107 (22.6)		398 (74.1)	66 (12.3)	73 (13.6)	
Meeting WHO recommendations	392 (52.0)	181 (24.0)	181 (24.0)		471 (70.6)	97 (14.5)	99 (14.8)	
Meeting recommendations for additional health benefit	370 (52.6)	146 (20.8)	187 (26.6)		301 (67.5)	64 (14.4)	81 (18.2)	
Self-reported health status; N, (%)				<0.0001				<0.0001
Very good	1,031 (53.8)	284 (14.8)	600 (31.3)		1,326 (73.4)	180 (10.0)	300 (16.6)	
Good	485 (40.7)	349 (29.3)	357 (30.0)		802 (66.3)	167 (13.8)	240 (19.9)	
Not so good	136 (31.8)	156 (36.5)	136 (31.8)		432 (75.4)	68 (11.9)	73 (12.7)	
Not good at all	52 (34.4)	54 (35.8)	45 (29.8)		186 (75.6)	29 (11.8)	31 (12.6)	

*According to the Global Recommendations on Physical Activity for Health. WHO publication. 2010. ISBN: 9789241599979

Table 4 shows variables found to be significantly and independently associated with past and current cigarette smoking, and Table 5 shows characteristics that were significantly and independently associated with a younger age at smoking initiation.

**Table 4:** Characteristics associated with smoking status in men and women: Multinomial logistic regression models. Data presented as odds ratio (95 % confidence limits); never smokers were the reference category

	Men		Women	
	Past smoking	Current smoking	Past smoking	Current smoking
Age group, yrs:				
20-24	1.00 (reference category)	1.00 (reference category)	1.00 (reference category)	1.00 (reference category)
25-34	2.15 (1.30- 3.54)	1.36 (0.99-1.87)	2.35 (1.27-4.37)	1.55 (1.06-2.27)
35-44	2.19 (1.27, 3.78)	1.13 (0.78-1.63)	2.58 (1.34-4.96)	1.42 (0.92-2.18)
45-54	4.32 (2.48-7.54)	1.61 (1.08-2.40)	2.16 (1.09-4.26)	1.53 (0.98-2.39)
55-64	6.74 (3.82-11.88)	1.23 (0.80-1.92)	2.33 (1.16-4.69)	1.14 (0.69-1.86)
65-74	7.44 (4.03-13.74)	0.82 (0.48-1.41)	3.16 (1.51-6.61)	0.70 (0.38-1.30)
75+	7.20 (3.75-13.84)	0.33 (0.17-0.65)	2.00 (0.88-4.55)	0.18 (0.08-0.42)
Population sub-group:				
Born in Israel	1.00 (reference category)	1.00 (reference category)	1.00 (reference category)	1.00 (reference category)
Immigrants before 1990	1.04 (0.79-1.37)	1.23 (0.92-1.65)	0.99 (0.72-1.37)	0.94 (0.68-1.30)
Immigrants between 1990 and 1995	1.18 (0.85-1.64)	1.37 (1.01-1.87)	0.55 (0.37-0.81)	0.71 (0.51-0.97)
Immigrants after 1995	1.66 (1.08-2.57)	1.23 (0.80, 1.88)	0.45 (0.26-0.78)	0.66 (0.43-1.01)
Arabs	0.74 (0.54-1.00)	1.53 (1.22, 1.93)	0.21 (0.12-0.36)	0.33 (0.24-0.47)
Years of education:				
0-8	1.00 (reference category)	1.00 (reference category)	1.00 (reference category)	1.00 (reference category)
9-12	0.81 (0.54-1.22)	0.69 (0.47-1.01)	1.43 (0.80-2.53)	1.17 (0.71-1.91)
13+	0.68 (0.46-1.01)	0.34 (0.23-0.50)	1.28 (0.73-2.24)	0.63 (0.38-1.03)
Religiosity:				
Secular	1.00 (reference category)	1.00 (reference category)	1.00 (reference category)	1.00 (reference category)
Traditional	0.86 (0.68-1.09)	1.02 (0.83-1.25)	0.67 (0.51-0.89)	0.90 (0.72-1.14)
Observant	0.72 (0.56-0.91)	0.60 (0.49-0.75)	0.37 (0.27-0.52)	0.39 (0.30-0.52)
Very religious	0.91 (0.61-1.35)	0.36 (0.23, 0.55)	0.24 (0.13-0.45)	0.06 (0.03-0.16)
Marital status:				
Married	1.00 (reference category)	1.00 (reference category)	1.00 (reference category)	1.00 (reference category)
Unmarried	1.01 (0.72-1.40)	1.22 (0.94-1.58)	1.00 (0.67-1.48)	2.45 (1.83-3.28)
Divorced	1.34 (0.85-2.01)	2.71 (1.79-4.11)	1.36 (0.93-2.00)	2.16 (1.56-2.99)
Widowed	0.83 (0.46-1.50)	1.62 (0.79-3.33)	1.11 (0.71-1.73)	1.85 (1.16-2.96)
Employment status:				
Employed	1.00 (reference category)	1.00 (reference category)	n.s.	n.s.
Unemployed	1.19 (0.70-2.03)	1.75 (1.17-2.60)		
Not included in the labor force	0.82 (0.62-1.08)	0.77 (0.59-1.00)		
Possession of assets:				
Neither car nor house	1.00 (reference category)	1.00 (reference category)	1.00 (reference category)	1.00 (reference category)
Car only	1.53 (1.02-2.29)	1.07 (0.77-1.47)	1.40 (0.89-2.19)	1.21 (0.83-1.76)
House only	1.04 (0.73-1.48)	0.66 (0.49-0.89)	0.57 (0.38-0.85)	0.56 (0.40-0.79)
A car and a house	1.10 (0.80-1.53)	0.59 (0.45-0.77)	0.65 (0.45-0.96)	0.69 (0.50-0.95)
A house and two cars or more	1.02 (0.67-1.55)	0.67 (0.47-0.94)	0.79 (0.49-1.28)	0.94 (0.63-1.40)
Two houses or more	1.24 (0.82-1.87)	0.75 (0.51-1.09)	0.89 (0.55-1.45)	0.61 (0.38-0.99)
Being successful in meeting living expenses:				
Unsuccessful	1.00 (reference category)	1.00 (reference category)	1.00 (reference category)	1.00 (reference category)
Not very successful	0.82 (0.55-1.22)	0.82 (0.58-1.15)	0.81 (0.50-1.30)	0.68 (0.47-0.97)
Successful	0.76 (0.52-1.11)	0.56 (0.40, 0.78)	0.66 (0.41-1.05)	0.42 (0.2- 0.61)
Highly successful	0.59 (0.39-0.90)	0.37 (0.25, 0.53)	0.56 (0.32-0.96)	0.68 (0.24-0.59)
Self-reported health status:				
Not good at all	1.00 (reference category)	1.00 (reference category)	1.00 (reference category)	1.00 (reference category)
Not so good	1.02 (0.63-1.64)	1.00 (0.60-1.68)	0.91 (0.52-1.57)	0.83 (0.48-1.45)
Good	0.65 (0.39-1.07)	0.84 (0.49-1.43)	1.11 (0.65-1.90)	1.23 (0.73-2.10)
Very good	1.26 (0.77-2.08)	1.16 (0.67-1.99)	0.77 (0.44-1.38)	0.82 (0.47-1.44)
Body mass index (kg/m^2^):				
Normal (<25.0)	1.00 (reference category)	1.00 (reference category)	1.00 (reference category)	1.00 (reference category)
Overweight (≥25.0 and <30.0)	1.11 (0.90-1.37)	0.97 (0.80-1.17)	1.34 (1.03-1.74)	0.98 (0.77-1.25)
Obese (≥30.0)	1.34 (1.02-1.75)	0.70 (0.53-0.92)	0.99 (0.70-1.38)	0.75 (0.55-1.01)
Self-reported leisure physical activity:*				
None	1.00 (reference category)	1.00 (reference category)	1.00 (reference category)	1.00 (reference category)
Less than recommended	1.05 (0.79-1.38)	0.62 (0.47-0.81)	0.93 (0.67-1.30)	0.64 (0.47-0.88)
Meeting WHO recommendations	0.62 (0.47-0.81)	1.02 (0.80-1.30)	1.08 (0.80-1.45)	0.67 (0.51-0.88)
Meeting recommendations for additional health benefit	0.92 (0.71-1.19)	0.64 (0.51-0.81)	1.21 (0.86-1.71)	0.86 (0.63-1.17)
Mean gross income per-capita per month:	n.s.	n.s.		
<2,000 NIS			1.00 (reference category)	1.00 (reference category)
2,000-4,000 NIS			1.21 (0.85-1.74)	1.43 (1.07-1.92)
>4,000 NIS			1.58 (1.09-2.29)	1.40 (1.08-1.93)
No information			1.62 (1.06-2.48)	1.17 (0.81-1.68)

n.s. : Non significant association

*According to the Global Recommendations on Physical Activity for Health. WHO publication. 2010. ISBN: 9789241599979

**Table 5:** Factors associated with younger age at smoking initiation among men and women*: Cox proportional hazard models

	Men	Women
	Hazard Ratio (95 % confidence interval)	Hazard Ratio (95 % confidence interval)
Birth cohort:		
1930-1949	1.00 (reference category)	1.00 (reference category)
1950-1959	1.09 (0.94-1.26)	1.02 (0.84-1.24)
1960-1969	1.12 (0.96-1.31)	1.17 (0.95-1.45)
1970-1979	1.13 (0.97-1.32)	1.53 (1.26-1.86)
1980-1989	1.53 (1.31-1.79)	2.21 (1.82-2.68)
Years of education:		n.s.
0-8	1.00 (reference category)	
9-12	1.02 (0.86-1.21)	
13+	0.86 (0.72-1.01)	
Religiosity:	n.s.	
Secular		1.00 (reference category)
Traditional		0.93 (0.80-1.07)
Observant		0.83 (0.70-0.99)
Very religious		1.40 (0.86-2.27)
Population sub-group:		n.s.
Born in Israel	1.00 (reference category)	
Immigrants before 1990	1.026 (0.888-1.185)	
Immigrants between 1990 and 1995	1.360 (1.165-1.586)	
Immigrants after 1995	1.178 (0.985-1.409)	
Arabs	0.849 (0.749-0.962)	

*Only Jewish women were included in the analysis (see methods section)

n.s.: Non significant association

We found an inverse association between year of birth and the age at smoking initiation, both among men and women. Men and women born between 1980 and 1989 were 1.5 and 2.2-times more likely to be younger at smoking initiation than men and women born between 1930 and 1949, respectively (Table 5).

Although Arab men were more likely to start smoking at an older age (Table 5), they were 1.5-times more likely to be current smokers than Israeli-born Jewish men (Table 4). In contrast, Arab women were 67 % less likely to report current cigarette smoking and about 80 % less likely to be past smokers than Israeli-born Jewish women (Table 4).

Within the Jewish population, men who immigrated to Israel in 1990 or thereafter were slightly more likely to be past or current smokers than men who were born in Israel (Table 4), and were significantly more likely to start smoking at a younger age (Table 5). In contrast, women who immigrated to Israel were significantly less likely to report past or current cigarette smoking compared to Israeli-born women (Table 4).

Men with 13 or more years of schooling had 66 % lower odds of being current smokers (Table 4), and had 14 % lower risk to start smoking at a younger age than men with 0-8 years of schooling (Table 5). Among women, the association between educational level and smoking status was less marked, and educational level was not significantly associated with age at smoking initiation in the multivariable regression model.

Religiosity was significantly and independently associated with smoking status in both gender groups. Observant and very religious participants, women especially, were less likely to report current cigarette smoking compared to secular men and women (Table 4). Women who defined themselves as observant were also less likely to start smoking at a younger age (Table 5).

We found a significant interaction between religiosity and ethnic group among men (*p* = 0.012). Although non-religious Arab men were more likely than non-religious Jewish men to be current smokers, the opposite was true among Arab men who defined themselves as traditional, religious, or very religious.

Divorced men were 2.7 times more likely to be current smokers than married men. Among women, being divorced, widowed or unmarried was associated with about two-times greater odds of being a current smoker compared to married women.

SES characteristics were strongly associated with smoking history among men and women. Unemployed men were 1.75 times more likely to report current smoking than employed men. Employment status was not found to be significantly associated with smoking status among women. Ownership of material assets and being successful in meeting living expenses were associated with lower likelihood of being a current smoker in both gender groups. We found a significant interaction between mean gross income per-capita per month and ethnic group among men (*p* = 0.003). Jewish women who immigrated to Israel and Arab women who reported a mean gross income per-capita < 2,000 NIS per month were less likely to report current smoking than Israeli-born Jewish women. In contrast, Jewish women who immigrated to Israel and Arab women who reported a mean gross income per-capita ≥ 4,000 NIS per month were significantly more likely to report current smoking than Israeli-born Jewish women.

Finally, being engaged in leisure physical activity was associated with lower odds of reporting current smoking both among men and women.

## Discussion

In the current study, we describe the association between smoking habits and a broad set of social characteristics in the ethnically diverse population of Israel.

We found that current smokers were less affluent than never smokers. Lower SES has been associated with higher smoking rates in other developed countries as well [8, 17, 18]. Multiple factors may explain the socio-economic gradient in smoking, including lower awareness of the health hazards of smoking, higher nicotine dependence and less supportive social environment [19]. We found that the association between smoking status and education or socio-economic parameters differed by gender. While unemployed men or men with lower educational level were more likely to be smokers, among women we found a positive association between cigarette smoking and income level, and the association between smoking status and educational levels was less clear. Similar gender-related differences in the association between income and educational level and smoking status have been reported in some southern European countries [20–22]. Possible explanations include failing of social barriers which traditionally prevented female smoking, association of female smoking with higher gender empowerment, using smoking for body weight control, and the effect of selective smoking advertising directed towards women [23–26].

We found that family status other than being married was associated with higher likelihood of current smoking, especially among women. Higher likelihood of current smoking among divorced and widowed people was reported also in the US population. The authors suggested that lack of social support may explain this finding [27]. Nevertheless, the greater likelihood of current smoking among single women found in our study suggests that other factors may also play a role, such as lack of incentives to stop smoking (e.g. no need to protect other family members sharing the same household from the ill effects of smoking).

Men who belong to the Arab minority group and male immigrants were more likely to be current smokers than Jewish men who were born in Israel, while opposite trends were found among women. The smoking rates among Arab men and women in our study were similar to those reported in other Middle-Eastern and North-African countries [28]. Gender difference in the association between ethnicity and smoking rates were also described in the African American and white population in the United States, where cigarette smoking was more common among African American men than among white men, while the opposite trend was observed among women [29]. Previous data from periodic telephone surveys in Israel showed that between 2000 and 2008 smoking rates decreased by 3.5 % among adult Jewish men, while an increase of 6.5 % was reported among Arab men [13]. A recent study also showed that Arab male smokers have low intention to stop smoking, with 60 % of current smokers being in the pre-contemplation phase [30].

Since the early 1990s, Israel has absorbed a large immigrant population from the former Soviet Union countries. The rates of current cigarette smoking among men in those countries is very high, ranging from 43 % in Moldova to 65 % in Kazakhstan, while cigarette smoking is relatively uncommon among women in these countries (2.4-15.5 %) [31].

The differences in the age of smoking initiation between Arabs, Israeli-born Jews and immigrants to Israel were previously described [14].

Despite a constant decline in cigarette smoking rates with increasing year of birth among men, there was a concomitant disturbing trend toward younger age at smoking initiation with increase in birth year in both gender groups. Our results confirm a previous report showing a trend of younger age at smoking initiation among young adults recruited to mandatory service in the Israel Defence Force [15]. Data from the US show that the age at smoking uptake remained stable across birth cohorts among men, while among women, the age at smoking initiation declined and is similar to men in recent birth cohorts [32].

This study is cross-sectional design, and thus one should be cautious in making inferences on causal associations. In addition, the information on smoking habits was based on self-reports and was not validated by testing cotinine blood or urine levels. Thus, possible differential misclassification by gender, ethnicity and other characteristics cannot be excluded. We also cannot exclude a recall bias related to age at smoking initiation, although the short-term reliability of this information tested in the Israeli population was good for both sexes [33]. However, the study sample, which was both large and representative, and the high consent rate provide high precision and external validity to our study results. The large set of social characteristics collected allowed us to study characteristics which are significantly and independently associated with smoking, after controlling for other closely related variables. With this respect, the current study differs from previous reports on smoking in Israel, that were either based on a selective population (e.g. soldiers recruited to mandatory military service in the Israel Defense Forces), or on data collected by land-line telephone interviews, with low consent rates. Such samples are prone to under-represent ultra-Orthodox Jews, Arabs, underprivileged populations, younger people and immigrants [13–15].

### Implications to health policy

#### Tobacco control

Evidence show that effective tobacco control measures in the form of tobacco products taxation, smoke-free legislation and tobacco sale restriction to minors is effective in reducing disparities associated with smoking uptake and promote smoking cessation [34, 35]. Although Israel has adopted such policies [36], ineffective or selective enforcement of smoke-free and sale restriction legislation impairs its efficacy and may in fact increase health disparities associated with cigarette smoking. In fact, selective enforcement may partially explain the opposite time trends of cigarette smoking rates observed in Jewish and Arab men [13]. Thus, to address disparities related to cigarette smoking, effective enforcement of smoke-free and sale restriction legislation should be directed at population subgroups with high smoking rates, for example Arab communities.

According to the law on compulsory reporting of tobacco smoking-related health damages, the Israel Minister of Health issues annual reports with comprehensive information on smoking rates, smoking cessation activities, and smoking prevention legislation/regulations [37]. These public domain reports are discussed in the Israeli parliament and media.

#### Smoking prevention/cessation

Targeting smoking prevention/cessation efforts at disadvantaged populations is considered a major means to reduce health disparities. Nevertheless, smoking cessation media campaigns were found less effective in motivating cessation attempts and smoking abstinence among people with lower educational level [38]. People from low-income groups were also found less likely to participate in smoking cessation interventions [39], to adhere to such interventions, to stop smoking and maintain long-term smoking abstinence compared to people of higher socioeconomic position [40, 41]. While economic incentives proved promising in motivating smokers to stop smoking, they were mostly implemented at worksites and in general included people from more advantaged groups [42, 43]. The feasibility and efficacy of such interventions among people who are socially disadvantaged have yet to be determined.

Culturally sensitive interventions targeted at minority adolescents in the US were effective in reduction of smoking uptake but not in increasing smoking cessation [44]. Recently, a feasibility study tested the effect of web-based intervention aimed to increase knowledge and reduce cigarette and nargila smoking among Arab university students, of whom one-fifth were smokers. The intervention was well-accepted, increased the proportion of students in the contemplation phase, but was not associated with cigarette smoking cessation after 1 month [45]. We are not aware of other studies evaluating the efficacy of smoking prevention/cessation interventions among low SES groups, immigrants and ethnic minority groups in Israel.

To reduce disparities related to cigarette smoking, smoking prevention/cessation programs should be culturally-congruent and address the different motives and needs among cigarette smokers who differ by ethnicity, immigration status, socioeconomic position, sex and cultural background. Smoking prevention/cessation programs and policy changes must be properly evaluated in these diverse population subgroups, in order to assess their effectiveness in reducing disparities related to cigarette smoking.

Systematic collection of up-to-date information on smoking history, cessation attempts and methods, integrated in the primary care electronic health record, using adequate alerts and decision rules, may be effective in increasing the proportion of smokers who receive advice to stop smoking and effective cessation intervention.

## Conclusions

There are various subgroups among the Israeli population where the uptake of cigarette smoking is high, namely: Arab minority men, men who are less affluent and who have lower educational levels, unmarried men and women and male immigrants. These population groups should be prioritized for intervention. We also found that smoking rates and age at smoking uptake differ by gender, cultural background and socioeconomic characteristics. These characteristics should be taken into account in the design and implementation of effective, culturally-congruent tobacco control and smoking prevention and cessation interventions. Further study is necessary to better understand causal pathways underlying the association between some of these social characteristics and smoking habits among various subgroups within the Israeli population. Finally, the current study was conducted in 2010, the same year that free-of-charge smoking cessation group sessions and subsidized smoking cessation medications were introduced in the Israeli health system. Further study is necessary to assess whether these measures are effective in reducing disparities associated with cigarette smoking in Israel.

**Table:** 

Summary Table
What is already known on disparities related to cigarette smoking in Israel
Cigarette smoking is more frequent among Arab men than among Jewish men. While the prevalence of smoking has declined among Jewish men and women in the past decade, an opposite trend was observed among Arab men.
The age of smoking initiation has declined over the past few decades. Younger age at smoking initiation is associated with male sex, lower educational level, lower socio-economic status and immigration from the former Soviet Union.
What this study adds
Marital status is associated with smoking rates; unmarried men and women are more likely to be current smokers.
Men and women with lower educational levels have greater odds for current smoking.
Observant or religious men and women are less likely to be ever-smokers.
The association between immigration and cigarette smoking differs by gender; while male immigrants are more likely to be current smokers than Israel-born Jewish men, the opposite is true with respect to women.
The association between some socio-economic characteristics and smoking also differ by gender; while unemployment is associated with higher likelihood of being a current smoker among men, higher mean gross income per capita is associated with higher odds for current smoking among women.

## Abbreviations

BMI, body mass index; DALYs, disability-adjusted life years; n.s., Non significant association (in figure only); SES, socioeconomic status

## Additional file

Additional file 1: Table S1. Correlation matrix* of the background variables by gender. (DOCX 19 kb)
