# Supplementary material for: Social characteristics associated with disparities in smoking rates in Israel
Source: Isr J Health Policy Res. 2016 Dec 1;5:36. doi: 10.1186/s13584-016-0095-2 (PMC5131466; doi:10.1186/s13584-016-0095-2)
Supplement: Additional file 1: Table S1. — Correlation matrix* of the background variables by gender. (DOCX 19 kb) [file 13584_2016_95_MOESM1_ESM.docx]

| Physical activity | BMI | SRH | Meeting living expenses | Possession of assets | Employment  status | Marital status | Religiosity | Years of education | Population sub-group | Age group |  |
| --- | --- | --- | --- | --- | --- | --- | --- | --- | --- | --- | --- |
| 0.09 | 0.22 | 0.31 | 0.09 | 0.15 | 0.39 | 0.43 | 0.06 | 0.25 | 0.26 |  | Age group |
| 0.11 | 0.07 | 0.21 | 0.12 | 0.15 | 0.16 | 0.15 | 0.17 | 0.25 |  | 0.26 | Population sub-group |
| 0.13 | 0.04 | 0.19 | 0.13 | 0.11 | 0.16 | 0.13 | 0.17 |  | 0.26 | 0.25 | Years of education |
| 0.07 | 0.04 | 0.05 | 0.08 | 0.15 | 0.16 | 0.09 |  | 0.17 | 0.21 | 0.07 | Religiosity |
| 0.10 | 0.20 | 0.16 | 0.07 | 0.12 | 0.14 |  | 0.11 | 0.18 | 0.21 | 0.46 | Marital status |
| 0.04 | 0.04 | 0.27 | 0.08 | 0.18 |  | 0.23 | 0.12 | 0.29 | 0.26 | 0.36 | Employment status |
| 0.09 | 0.09 | 0.12 | 0.13 |  | 0.17 | 0.19 | 0.14 | 0.16 | 0.17 | 0.18 | Possession of assets |
| 0.11 | 0.07 | 0.12 |  | 0.15 | 0.06 | 0.08 | 0.09 | 0.09 | 0.08 | 0.11 | Meeting living expenses |
| 0.13 | 0.14 |  | 0.12 | 0.18 | 0.28 | 0.25 | 0.08 | 0.23 | 0.24 | 0.37 | SRH |
| 0.08 |  | 0.23 | 0.09 | 0.09 | 0.13 | 0.17 | 0.04 | 0.11 | 0.14 | 0.26 | BMI |
|  | 0.06 | 0.12 | 0.08 | 0.09 | 0.08 | 0.07 | 0.08 | 0.16 | 0.12 | 0.08 | Physical activity |
| 0.11 | 0.06 | 0.11 | 0.20 | 0.19 | 0.22 | 0.12 | 0.24 | 0.19 | 0.20 | 0.15 | Income |

Additional file 1: Table S1: Correlation matrix* of the background variables by gender

*-The correlation between variables was tested using the Cramér's V statistic, which is a measure of association between two nominal variables, giving a value between 0 and +1. Values for men are marked in blue and for women in red.

SRH: self-rated health status;

BMI: body mass index;
